# Identification of a two-marker-haplotype on Bos taurus autosome 18 associated with somatic cell score in German Holstein cattle

**DOI:** 10.1186/1471-2156-10-50

**Published:** 2009-09-02

**Authors:** Bodo Brand, Christine Baes, Manfred Mayer, Norbert Reinsch, Christa Kühn

**Affiliations:** 1 Research Unit Molecular Biology, Research Institute for the Biology of Farm Animals, 18196 Dummerstorf, Germany; 2 Research Unit Genetics and Biometry, Research Institute for the Biology of Farm Animals, 18196 Dummerstorf, Germany

## Abstract

**Background:**

The somatic cell score (SCS) is implemented in routine sire evaluations in many countries as an indicator trait for udder health. Somatic cell score is highly correlated with clinical mastitis, and in the German Holstein population quantitative trait loci (QTL) for SCS have been repeatedly mapped on *Bos taurus *autosome 18 (BTA18). In the present study, we report a refined analysis of previously detected QTL regions on BTA18 with the aim of identifying marker and marker haplotypes in linkage disequilibrium with SCS. A combined linkage and linkage disequilibrium approach was implemented, and association analyses of marker genotypes and maternally inherited two-marker-haplotypes were conducted to identify marker and haplotypes in linkage disequilibrium with a locus affecting SCS in the German Holstein population.

**Results:**

We detected a genome-wide significant QTL within marker interval 9 (*HAMP_c.366+109G>A *- *BMS833*) in the middle to telomeric region on BTA18 and a second putative QTL in marker interval 12-13 (*BB710 *- *PVRL2_c.392G>A*). Association analyses with genotypes of markers flanking the most likely QTL positions revealed the microsatellite marker *BMS833 *(interval 9) to be associated with a locus affecting SCS within the families investigated. A further analysis of maternally inherited two-marker haplotypes and effects of maternally inherited two-marker-interval gametes indicated haplotype *249-G *in marker interval 12-13 (*BB710 *- *PVRL2_c.392G>A*) to be associated with SCS in the German Holstein population.

**Conclusion:**

Our results confirmed previous QTL mapping results for SCS and support the hypothesis that more than one locus presumably affects udder health in the middle to telomeric region of BTA18. However, a subsequent investigation of the reported QTL regions is necessary to verify the two-QTL hypothesis and confirm the association of two-marker-haplotype *249-G *in marker interval 12-13 (*BB710 *- *PVRL2_c.392G>A*) with SCS. For this purpose, higher marker density and multiple-trait and multiple-QTL models are required to narrow down the position of the causal mutation or mutations affecting SCS in German Holstein cattle.

## Background

Udder health, somatic cell score and subclinical and clinical mastitis remain major challenges for the economy of milk production in respect to milk production efficiency and animal health and welfare. Several studies have attempted to identify chromosomal regions, genes and polymorphisms that influence udder health in order to improve breeding strategies. SCS has been used as an indicator of udder health and is implemented in routine sire evaluations in many countries [1]. SCS has a low to medium heritability (h^2 ^= 0.15; [1]) and a strong correlation to mastitis in the German Holstein population (r_g _= 0.84; [2]). However, selection on low SCS as well as on decreased mastitis incidence is hampered by three aspects: first the low heritability of SCS and liability to mastitis, second the difficulties in recording mastitis related data and third by potential population-wide antagonisms between milk production traits (milk, fat and protein yield) and udder health [1,2]. Recently, marker assisted selection (MAS) has been determined as a promising tool to improve current selection strategies based on phenotypic data [3]. MAS implements genetic marker information of confirmed QTL regions to identify individuals with favourable genetic background concerning the trait of interest. Thus, the confirmation and fine mapping of known QTL regions and the estimation of QTL effects will advance the use of MAS.

For clinical mastitis (CM) or SCS, QTL have been detected on nearly all autosomes [4] and several studies repeatedly detected QTL for SCS or CM in the telomeric region of BTA18 [5-12]. In addition, Kühn et al. [13] have shown in a proof-of-principle approach that information of 5 markers located in the telomeric region of BTA18 indeed enabled successful MAS, which identified halfsib heifers prior to first calving that exhibited significant differences in SCS after parturition.

The intention of this study was to further analyse the middle to telomeric region of BTA18 with the aim of identifying markers and marker haplotypes in linkage disequilibrium (LD) with SCS in German Holstein cattle to improve MAS for udder health. Therefore, we increased the marker density in the telomeric region on BTA18 and selected four functional candidate genes within the QTL regions reported by [5,7,11]. Polymorphisms detected within these candidate genes were used as additional markers for fine mapping previously identified QTL regions and to analyse effects of candidate gene polymorphisms on SCS in the German Holstein. In the present study, we detected a genome-wide significant QTL in the middle to telomeric region on BTA18. Furthermore, we analysed effects of maternally inherited marker haplotypes and identified a two-marker-haplotype associated with SCS in German Holstein cattle.

## Methods

### Selection of candidate genes

Based on the positional information derived from the previously mentioned QTL regions identified by [5,7,11] and preliminary results from microarray experiments, an intensive literature research of genes bearing potential function in innate immune defence, immune defence, mammary gland development or udder morphology was performed. To enable the investigation of positional candidate genes within the telomeric region on BTA18, a comparative map for BTA18 and *Homo sapiens *autosome 19 (HSA19) was constructed to take advantage of the more advanced gene annotation of the human genome (Additional file 1: marker table). Four candidate genes were selected: *calmodulin 3 *(phosphorylase kinase, delta) (*CALM3*), *Hepcidin antimicrobial peptide *(*HAMP*), *cadherin 1, type 1, E-Cadherin *(ephitelial) (*CDH1*), and *poliovirus receptor-related 2 *(herpesvirus entry mediator B) (*PVRL2*). Calmodulin 3 is a ubiquitously expressed Ca^2+^-binding protein that is involved in many Ca^2+ ^modulated signal pathways. It was selected due to its influence on smooth muscle contraction [14,15] and the potential effects on milk leaking and milk flow, which is a trait with a substantial genetic correlation to SCS (r_g _= 0.4; [1]). *HAMP *was selected as an immunological candidate gene because of its antifungal and antibacterial activity [16], the toll-like receptor-4 dependent induction by bacterial pathogens in myeloid cells [17] and to a lesser extent due to its function as a key regulator of iron metabolism [18]. For *CDH1 *and *PVRL2 *initial results from own microarray experiments indicated their differential expression in clinically unaffected heifers with different predisposition to udder infection. Additionally, both genes are involved in cell-cell junctions that have a strong impact on tissue development. E-Cadherin, a Ca^2+^-dependent cell-cell adhesion molecule, is involved in tissue and organ development as part of the cadherin-catenin-complex [19,20], and poliovirus receptor-related 2, a Ca^2+^-independent immunoglobulin-like cell adhesion molecule, is involved in the organisation of intercellular junctions as part of the nectin-afadin-complex [20-22]. Furthermore E-Cadherin can serve as a receptor for pathogens and is involved in their internalization [23,24].

### Screening for polymorphisms

Polymorphisms within the candidate genes were detected by comparative sequencing of genomic DNA from heifers selected for QTL alleles associated with high or low SCS. The heifers originate from most likely QTL heterozygous sires selected from the German Holstein population based on marker information regarding a confirmed QTL for SCS on BTA18. The selection strategy for the heifers and their phenotypes are described by Kühn et al. [13] in detail. For sequencing, we selected three pairs of halfsibs, where one daughter inherited the SCS increasing paternal chromosomal region (q) and the other inherited the SCS decreasing paternal chromosomal region (Q). In addition, two animals were screened for variants which originated from a genetically divergent Charolais × Holstein F_2 _cross background.

For each gene except *CDH1 *the entire gene (large introns excluded), 800 to 1500 bp of the promoter region and up to 500 bp downstream of the transcripts were investigated. For *CDH1*, only the genomic sequence spanning exon 13 to 15 was analysed. Primer information and the genomic position for each primer are given in additional file 2: primer table sequencing. Sequencing was performed by amplification of genomic DNA and subsequent sequence analysis with the DYEnamic ET Terminator Cycle Sequencing reaction and the MegaBACE™1000 DNA Analysis System (GE Healthcare, Munich, Germany). For evaluation of polymorphisms BioEdit 7.0.5.2 [25] was used. Essentially, polymerase chain reaction (PCR) primers were used for sequencing. Additional sequencing primers were used only for longer PCR fragments or PCR fragments that were difficult to sequence.

### Families

The pedigree material used for genotyping included a total of 1,054 animals originating from six paternal halfsib families. Some of the animals are a subset of the granddaughter designs previously described by [7,11]. Numbers of sons per grandsire ranged from 60 to 353, with an average family size of 175 sons. The German genetic evaluation center (VIT) in Verden, Germany provided additional pedigree information including non-genotyped ancestors of genotyped animals (7,627 animals).

### Phenotypes

The phenotype information for SCS was provided by the VIT as daughter yield deviations (DYD) for the first lactation (Table 1). SCS is the log_2 _transformed somatic cell count (log_2 _(somatic cell count/100000) + 3). DYD for SCS were calculated based on a random regression test day model [26]. The reliabilities associated with the DYD were expressed as the number of effective daughter contributions (EDC) as described by [26]. DYD and EDC for genotyped animals were obtained from the official release of the April 2008 routine genetic evaluation.

**Table 1:** Descriptive statistics for daughter yield deviations

**Trait**	**Number of Phenotypes**	**Mean**	**Standard Deviation**	**Minimum**	**Maximum**
SCS	1058	- 0.1024	0.383	-1.211	1.072

Arithmetic mean and standard deviation as well as lowest and highest daughter yield deviation for SCS considering all animals are given. SCS is the log_2 _transformed somatic cell count (log_2 _(somatic cell count/100000) + 3).

### Marker Set

The marker set included a total of 28 markers covering the telomeric region of BTA18 from *CDH1 *to *DIK4013*. Six of the 28 markers were already genotyped within previous QTL mapping studies [5,7,11] and include an erythrocyte antigen marker. The other 22 markers were newly selected and genotyped. Fifteen new microsatellite and seven new single nucleotide polymorphism (SNP) markers were chosen based on the information of the putative QTL positions reported by [5,7,11]. The fifteen new microsatellite markers were selected using the bovine linkage map of the United States Department of Agriculture's Meat Animal Research Center (MARC USDA) [27]. SNP markers were selected from all detected polymorphisms within candidate genes based on allele frequencies, position (coding- or non-coding region), effect of the SNP (synonymous or nonsynonymous) and whether they are in linkage disequilibrium to each other.

### Genotyping

Microsatellite markers were genotyped by PCR or Multiplex-PCR with fluorescence labelled primers followed by a fragment length analysis using the MegaBACE™1000 DNA Analysis System and MegaBACE Fragment Profiler Version 1.2 software (GE Healthcare, Munich, Germany) (Additional file 3: primer table genotyping). The genotyping methods used for detection of SNP were PCR-restriction fragment length polymorphism (RFLP) assays for *CDH1_c.2102C>T *[NCBI dbSNP: rs41862198], *HAMP_c.366+109G>A *[NCBI dbSNP: ss141026745], *PVRL2_c.-1268G>C *[NCBI dbSNP: ss141026747], *PVRL2_c.392G>A *[NCBI dbSNP: rs41884977], *CALM3_c.3+1795C>T *[NCBI dbSNP: ss141026788] and a multiplex pyrosequencing assay for *HAMP_c.86+430G>A *[NCBI dbSNP: ss141026740] and *CALM3_c.3+1678C>T *[NCBI dbSNP: ss141026787] (Table 2). The enzymes used for detection of RFLPs were identified with the NEBcutter V2.0 webtool [28,29] and PCR primers were designed with primer analysis software Oligo 4.1 (National Bioscience Inc., Plymouth, MN, USA). For the RFLP assays, a PCR specific for each SNP was used to amplify genomic DNA, and PCR products were subsequently incubated for 8 h with SNP specific restriction enzymes. For visualization of the RFLP a 2.5% agarose gel was used.

**Table 2:** Primer and enzymes used for genotyping of SNP

**SNP**	**Method**		**Primer**	**Enzyme**	**Alleles**
*CDH1_c.2102C>T*	RFLP	forward	5'-CAT AGA CAA CCA GAA CAA AGA C	*Eco*57I	A/G
		reverse	5'-TGG ACC TCT GGG GAG ACT G	(Fermentas Life Sciences)	
*HAMP_c.366+109G>A*	RFLP	forward	5'-AGA CAC CCA CTT TCC CAT C	*Csp*6I	A/G
		reverse	5'-AGC TCC ACA GTC TCT TCT C	(Fermentas Life Sciences)	
*PVRL2_c.-1268G>C*	RFLP	forward	5'-AAT GCC AGT CAA TCA CAG TCT C	*Hinc*II	G/C
		reverse	5'-GGA TTC TAC ACC CGC TGC TC	(Fermentas Life Sciences)	
*PVRL2_c.392G>A*	RFLP	forward	5'-TTC CTC AAA CTG TCT TAT CTG G	*Hin*6I	A/G
		reverse	5'-GTG TAG TTG CCC TCG TCC TC	(Fermentas Life Sciences)	
*CALM3_c.3+1795C>T*	RFLP	forward	5'-TTG AGA GAA AAC CAG CAG AC	*Bse*YI	C/T
		reverse	5'-CCA GGC AGC AGT GTT AGA	(New England Biolabs Inc.)	
*CALM3_c.3+1678C>T*	pyro-sequencing	forward	5'-GAG CCC TCC CTG AGT GCT TC		C/T
		reverse	5'-AGC GGC TGC CTG TTC TCC		
		sequencing	5'-AGG ATG GCT GCA CAC		
*HAMP_c.86+430G>A*	pyro-sequencing	forward	5'-AAA AGA TGG TGG GAG AGT AAT GG		A/G
		reverse	5'-CCT CTG CAC TTG CCT GTA AGA CTT		
		sequencing	5'-CCA AAT AGG TCA AAT AAC A		

Summary of methods and primers used for genotyping SNP. Forward primers for pyrosequencing were biotyinlated at the 5'-end for purification of single strand DNA.

Primer design for pyrosequencing was performed with Pyrosequencing™ Assay Design Software (Biotage AB, Uppsala, Sweden). A SNP specific PCR was used for amplification of genomic DNA and the products of both PCR were merged and analysed with the PSQ™HS 96A pyrosequencing system (Biotage AB, Uppsala, Sweden) in a multiplex run.

### Linkage Map

The genetic linkage map was calculated based on a refined marker order using CRIMAP software [30]. The marker order on BTA18 used for the calculation was evaluated in two steps. First, information from published linkage maps [31,32], RH-maps [33], and human and bovine sequence-assemblies [34] were merged and compared to own linkage-mapping results. Second, in a region including the markers *BMON117*, *DIK4672*, *BMS833*, *DIK4232 *and *BB710*, no unequivocal marker order was obtained. Thus, the marker order in this region was verified by RH-mapping using the 12000 rad whole-genome radiation hybrid panel [35] and RH-MAP3.0 software [36]. For some marker groups, a recombination rate of zero was calculated. To avoid technical difficulties arising in the calculation of transmitting probabilities the marker spacing was set to small values greater than zero (Additional file 1: marker table).

### QTL Mapping

A combined linkage and linkage disequilibrium analysis (LALD) was performed using the software system TIGER [37]. TIGER is a UNIX script linking several individual Fortran programmes to perform combined linkage and linkage disequilibrium analysis. Six steps are implemented in the script. First, allele frequencies and transmitting probabilities for each putative QTL position are calculated using BIGMAP [38]. The putative QTL positions were considered as the midpoint of each marker interval, resulting in a total of 27 putative QTL positions. Second, the identical by descent (IBD) sub-matrices for each putative QTL position are computed based on the gene dropping procedure described by [39-41] and third, the IBD sub-matrices are tested for positive definiteness and inverted. The software program COBRA [42] computes a condensed gametic relationship matrix and its inverse at each putative QTL position for the calculation of gametic effects. Transmitting probabilities and IBD sub-matrices are used for the set up of the condensed gametic relationship matrix. Finally, an LALD analysis is performed analysing every putative QTL position with restricted maximum likelihood (REML) methods applied in ASReml [43]. A detailed description of the QTL mapping procedure and the model applied in ASReml is given by [12].

Analyses were conducted using a likelihood ratio test, where the REML of the full model was compared with the REML of the model missing the QTL effect. Chromosome-wide and genome-wide significance thresholds were determined as restricted log likelihood ratio (RLRT) equivalents of logarithm of odds (LOD) units [44] where a LOD > 2 indicates chromosome-wide (RLRT = 9.2) and a LOD > 3 indicates genome-wide significance (RLRT = 13.8) [45]. Confidence intervals were estimated using the LOD drop-off method described by [44].

### Association analysis

To investigate the association of candidate gene polymorphisms and markers flanking interval 9 and interval 12-13 with SCS, a mixed model including a random polygenic effect and the fixed effect of marker genotypes was applied in ASReml:

(1)

where **y **is a vector of phenotypic observations (DYD) for sires, **μ **is the overall mean, **MG**_i _is the fixed effect of the marker genotype **i**, **a**_j _is the random polygenic effect of animal **j **and **e**_ij _is the random residual. The polygenic effect that accounts for the family structure of the population was estimated using an extended pedigree of non-genotyped ancestors of genotyped animals including a total of 7,627 animals. To account for multiple testing, a 5% experiment-wise significance threshold was obtained by Bonferroni correction of the nominal p-value assuming a 5% Type 1 error (p_exp _= 0.0057).

### Analysis of maternally inherited two-marker haplotypes and two-marker-interval gametes

Due to the limited number of sires, paternally inherited chromosomes could have a strong impact on genotype effects estimated in their offspring. To exclude these effects, maternally inherited two-marker-intervals including flanking markers of most likely QTL position were investigated. The most probable linkage phases of genotyped sires were calculated to determine the maternally inherited haplotypes using BIGMAP. The TIGER software system was then applied to estimate maternally inherited gametic effects for the putative QTL positions in interval 9 and interval 12 based on IBD sub-matrices. Finally, the estimated gametic effects of maternally inherited two-marker-interval gametes were analysed and plotted using SAS software (SAS Institute, Cary, NC, USA).

To verify differences in estimated two-marker-interval gamete effects, we performed an association analysis including a fixed maternally inherited two-marker-haplotype effect independent of the IBD sub-matrices. For this purpose, the same model was used as for marker genotypes (1), except that the fixed genotype effect was replaced by the fixed maternally inherited two-marker-haplotype effect. Two-marker-haplotypes with the highest and lowest mean for IBD gametic effects were tested against the total of all other haplotypes within respective intervals. For interval 12-13 additionally the most frequent two-marker-haplotype was investigated.

## Results

### Screening for polymorphisms

Sequence analyses were based on the provisional reference sequences obtained from NCBI [46]. For *CALM3 *[GenBank: NM_001046249; GeneID: 520277], the entire gene (10728 bp) was resequenced including ~1.3 kb upstream from the transcription start (assumedly promoter region). A total of eleven polymorphisms were detected (Additional file 4: polymorphisms): ten SNP and one 12 bp deletion within the assumed promoter region.

For *HAMP *[GenBank: NM_001114508; GeneID: 512301], in silico analyses revealed that this gene is not annotated in Btau4.0 but has been annotated in NCBI Build3.1. The sequence of *HAMP *is still located on BTA18 in Btau4.0 [GenBank: NC_007316.3: 476114 bp-477570 bp]. The whole gene (2777 bp) was resequenced, and three SNP were detected (Additional file 4: polymorphisms), one SNP within intron 1 and two SNP within the first 110 nucleotides downstream of *HAMP *transcript.

For *CDH1*, only the coding sequence was provided as provisional mRNA reference sequence [GenBank: NM_001002763; GeneID: 282637]. Due to discrepancies between the mRNA reference sequence and the genome assembly sequence, only the genomic reference sequence was used for sequence comparisons. The genomic sequence including exon 13 to 15 (1673 bp) was investigated and six SNP were detected (Additional file 4: polymorphisms) two of them were located in exon 13 and cause an amino acid substitution.

For *PVRL2 *[GenBank: NM_001075210.1; GeneID: 505580], the whole gene, excluding 13181 bp of intron 2 and 670 bp of intron 4, was resequenced (11766 bp). A total of 17 SNP and two length polymorphisms were identified (Additional file 4: polymorphisms). The examination of the promoter region (1 kb upstream of transcription start) of *PVRL2 *revealed a gap of 367 bp with missing sequence information in the genome assembly Btau4.0. By a BLAST search [47] a whole genome shotgun sequence (WGS)-Trace [Trace Archive: 1632871820] that overlaps the gap was identified within the NCBI Trace Archives [48]. This WGS-Trace was incorporated in the reference sequence and sequence information was confirmed by resequencing. Coordinates of polymorphisms upstream of the translation initiation codon ATG are indicated according to the updated sequence [GenBank: FJ829796].

### Markers and Map

The genetic marker map covered 50 cM of the telomeric region of BTA18 (Additional file 1: marker table). Marker intervals ranged from 0.05 cM to 7.9 cM with an average marker interval of 1.85 cM. The number of alleles for each marker ranged from two for SNP to 34 for the erythrocyte antigen marker. The marker order is in good agreement with previously published linkage- and RH-maps [31-33] and no discrepancies to the bovine sequence assembly Btau4.0 [49] were observed (Additional file 1: marker table).

### QTL Mapping

**Figure 1:**
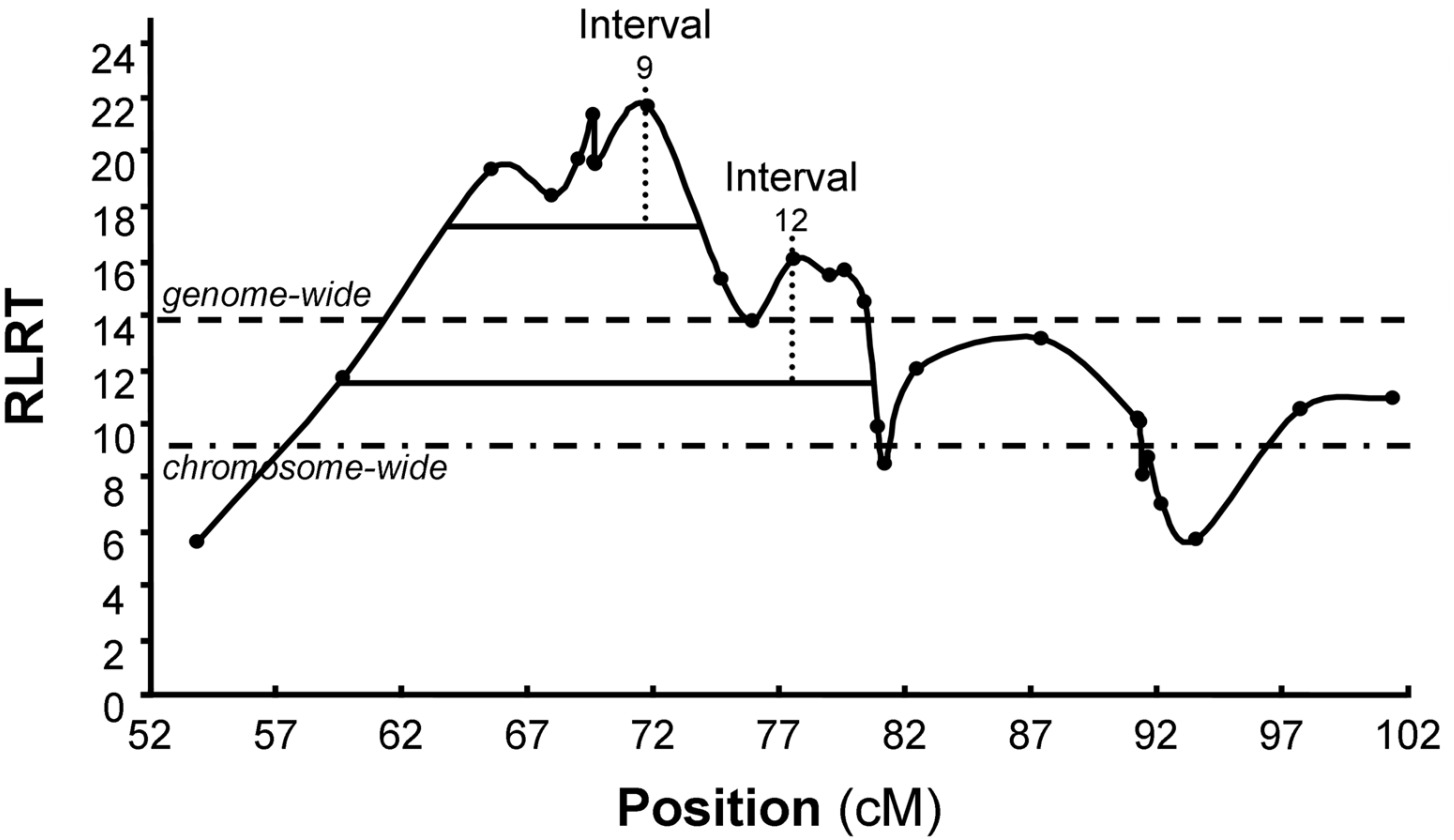
**LALD Profile**. Restricted Log Likelihood Ratio (RLRT) profile of a combined linkage and linkage disequilibrium analysis testing for a putative QTL affecting SCS on BTA18. The profile is plotted for putative QTL positions located at the midpoint of each marker interval. QTL positions are indicated by black dots. Thick black lines indicate confidence interval for the first maximum at interval 9 (upper line) and the second maximum at interval 12 (lower line). Dashed lines indicate genome- and chromosome-wide significance thresholds.

The restricted Log Likelihood Ratio profile of the combined LALD analysis is shown in Figure 1. Two peaks exceeding the genome-wide significance level can be observed. The maximum of the first peak is located in marker interval 9 at position 71.775 cM with *HAMP_c.366+109G>A *and *BMS833 *as flanking markers. The maximum of the second peak is located in marker interval 12 at position 77.6 cM with the flanking markers *BB710 *and *PVRL2_c.-1268G>C*. The LOD drop-off method was used to estimate confidence intervals for each of the peaks. An approximate 96% confidence interval included marker intervals 3 to 9 for the first peak and the marker intervals 2 to 15 for the second peak.

### Association analysis

An association analysis between the candidate gene polymorphisms and SCS was performed to evaluate the influence of the selected candidate genes on the variation in SCS in German Holstein cattle. The markers *BMS833 *and *BB710 *were also included in the association analysis, as they are flanking markers of the maxima observed in the LALD test statistic. The association analyses (Table 3) indicated a significant effect of the *BMS833 *genotype (p = 0.004) on SCS within the 5% experiment-wise significance threshold (p_exp _= 0.0057). *PVRL2_c.392G>A *(p = 0.017) and *CALM3_c.3+1678C>T *(p = 0.055) approached nominal significance, but were not significant at the 5% experiment-wise significance level. For the genotypes of SNP markers *CDH1_c.2102C>T*, *HAMP_c.366+109G>A*, *HAMP_c.86+430G>A*, *PVRL2_c.-1268G>C *and *CALM3_c.3+1795C>T *no significant effects on SCS were observed.

**Table 3:** Association analyses of marker genotypes with SCS in six German Holstein halfsib families

**Marker/SNP**	**Position in cM**	**Nominal p-value**
*CDH1_c.2102C>T*	52	0.121
*HAMP_c.86+430G>A*	69.7	0.132
*HAMP_c.366+109G>A*	69.75	0.416
*BMS833*	73.8	0.004*
*BB710*	76.3	0.216
*PVRL2_c.-1268G>C*	78.9	0.325
*PVRL2_c.392G>A*	79.1	0.017
*CALM3_c.3+1678C>T*	81.15	0.055
*CALM3_c.3+1795C>T*	81.25	0.223

Summary of results from association analyses for marker genotypes based on a mixed model (1) including a fixed genotype effect and a random polygenic effect. The experiment-wise 5% significance level is p_exp _= 0.0057.

### Analysis of maternally inherited two-marker haplotypes and two-marker-interval gametes

**Figure 2:**
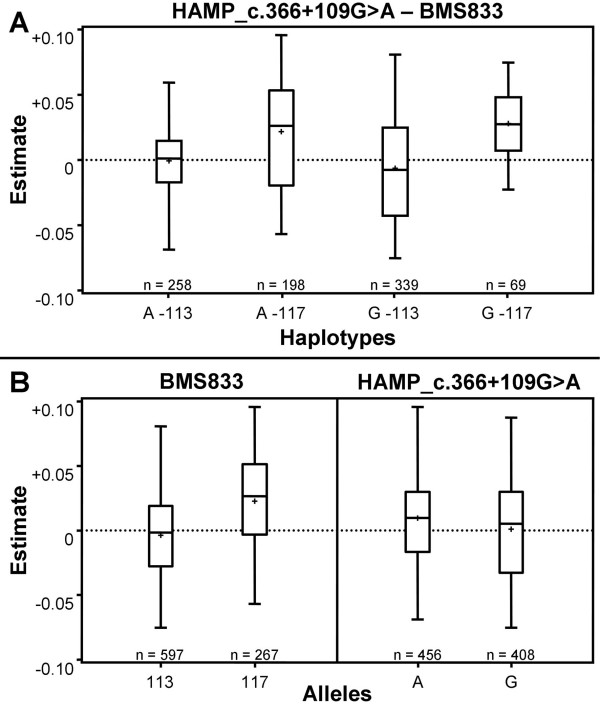
**Box-whisker plot for estimated effects of maternally inherited two-marker-interval gametes in interval 9**. Two-locus-interval (A) and single locus (B) gametes of the two-marker-interval flanking the putative QTL in interval 9 are written on the X-axis. The boxes contain 50% of all values, where (+) represents the mean, and the horizontal line within the box (-) represents the median. The first and third quartiles are represented by the lower and upper edge of the box and the whiskers extend to the highest and lowest values.

To exclude any specific sire gamete effects that occur in a paternal halfsib design, maternally inherited gametic effects for two-marker-interval gametes were analysed and plotted for the putative QTL positions in interval 9 and interval 12-13. In interval 9 (*HAMP_c.366+109G>A *- *BMS833*), six maternally inherited two-marker-allele combinations occurred. Two-marker-interval gametes including *BMS833 *alleles *115 *and *119 *were excluded, because they both occurred only once (Table 4; Figure 2A). Gametes carrying the *BMS833 *allele *117 *in interval 9 have a mean estimated maternally inherited gametic effect of 0.022 (± 0.003) and 0.028 (± 0.003), respectively, whereas gametes carrying allele *113 *of marker *BMS833 *have mean effects of - 0.0006 (± 0.0014) and - 0.0059 (± 0.0022), respectively. Investigating the gametic effect of each flanking marker of interval 9 alone (Figure 2B), revealed that the main differences in gametic effects in the two-marker-interval results from the discrimination by microsatellite marker alleles. The difference in the mean effects for two-marker-interval gametes carrying the two alleles of *HAMP_c.366+109G>A *is 0.009, whereas the difference between gametes carrying alleles *113 *and *117 *of marker *BMS833 *is 0.0268.

**Table 4:** Estimated effects on SCS for maternally inherited two-marker-gametes in interval 9 and interval 12-13

**Interval**	**Haplotype**	**Number of Animals**	**Haplotype Frequencies**	**Mean of Estimates**	**Standard Error Mean**
9	*A - 113*	258	0.2986	-0.0006	0.0014
9	*A - 117*	198	0.2292	0.0217	0.003
9	*G - 113*	339	0.3924	-0.0059	0.0022
9	*G - 117*	69	0.0799	0.0276	0.003

12 - 13	*249 - G*	38	0.0409	0.0378	0.0018
12 - 13	*249 - A*	262	0.2820	0.0176	0.0009
12 - 13	*251 - G*	78	0.0840	-0.0012	0.0007
12 - 13	*251 - A*	32	0.0344	0.0002	0.0024
12 - 13	*253 - G*	3	0.0032	-0.0150	0.0021
12 - 13	*253 - A*	44	0.0474	-0.0253	0.0012
12 - 13	*255 - G*	53	0.0571	0.0122	0.0009
12 - 13	*255 - A*	375	0.4037	-0.0043	0.0009
12 - 13	*257 - G*	4	0.0043	-0.0019	0.0022
12 - 13	*257 - A*	40	0.0431	0.0144	0.0022

Summary of means for gametic effects estimated for maternally inherited two-marker-interval gametes considering IBD coefficients. Haplotype frequencies were determined by direct counting.

**Figure 3:**
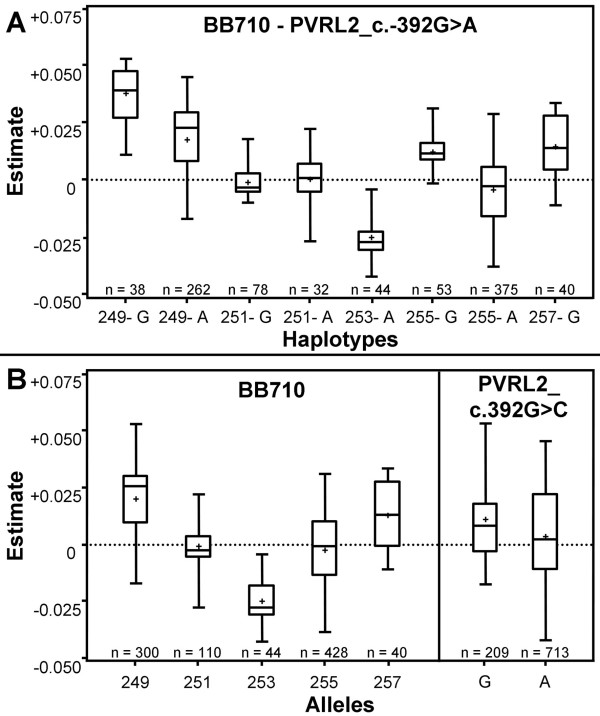
**Box-whisker plot for estimated effects of maternally inherited two-marker-interval gametes in interval 12-13**. Two-locus-interval (A) and single locus (B) gametes of the two-marker-interval *BB710 *(interval 12) and *PVRL2_c.392G>A *(interval 13) are written on the X-axis. Estimates are for the putative QTL position in interval 12. The boxes contain 50% of all values where (+) represents the mean and the horizontal line within the box (-) represents the median. The first and third quartiles are represented by the lower and upper edge of the box and the whiskers extend to the highest and lowest values.

For interval 12-13 (*BB710 *- *PVRL2_c.392G>A*) we selected *PVRL2_c.392G>A *(interval 13) as a flanking marker. Both polymorphisms, *PVRL2_c.-1268G>C *(interval 12) and *PVRL2_c.392G>A *(interval 13) are located in close vicinity within the *PVRL2 *gene and showed a high linkage disequilibrium (r^2 ^= 0.68). In interval 12-13 (*BB710 *- *PVRL2_c.392G>A*), two-marker-interval gametes with twelve different two-marker-allele combinations were observed. Two-marker-interval gametes carrying the two *BB710 *alleles *243 *and *245 *were excluded, because they both only occurred once. In addition, the frequencies for allele combinations *253-G *and *257-G *were smaller than 1% and therefore gametes carrying these two allele combinations are not included in Figure 3 (Table 4). Analogous to the situation in interval 9, the SNP alleles of *PVRL2_c.392G>A *seem to have only a small influence on discriminating estimated two-marker-interval gamete effects (Figure 3B), because the difference in the mean effects discriminated by the alleles is 0.0078. For microsatellite marker alleles (Figure 3A), two-marker-interval gametes carrying the allele *249 *have mean effects of 0.0378 (± 0.0018) and 0.0176 (± 0.0009) and gametes carrying allele *253 *have mean effects of - 0.0150 (± 0.0021) and - 0.0253 (± 0.0012). The biggest differences in the mean estimated gametic effects was observed for the two two-marker-interval gametes *249-G *(0.0378 (± 0.0018)) and *253-A *(-0.0253 (± 0.0012)). Thus, the difference in the mean maternally inherited gametic effects equals 0.0631, which is equivalent to 0.16 phenotypic standard deviations.

To validate the differences observed between maternally inherited two-marker-interval gametes, we performed a direct association analysis for maternally inherited two-marker-haplotypes without considering IBD coefficients in interval 9 (*HAMP_c.366+109G>A *- *BMS833*) and interval 12-13 (*BB710 *- *PVRL2_c.392G>A*) (Table 5). Only the two-marker-haplotypes with the highest and lowest mean for IBD gametic effects were tested against the total of all other haplotypes. For interval 12-13, we also tested *255-A*, because it was the most frequent haplotype (frequency at 40%) in the data set. In interval 9, no significant association of the maternally inherited haplotypes *G-113 *and *G-117 *were observed, whereas haplotype *249-G *(p = 0.027) in interval 12-13 seems to be associated with SCS in the German Holstein population.

**Table 5:** Association analyses of maternally inherited two-marker-haplotypes with SCS

**Interval**	**Haplotype**	**Haplotype Frequencies**	**Nominal p-value**	**Effect Estimates**
9	*G - 113*	0.3924	0.555	-0.016
9	*G - 117*	0.0799	0.422	0.038

12 - 13	*249 - G*	0.0409	0.027*	0.142
12 - 13	*253 - A*	0.0474	0.207	-0.073
12 - 13	*255 - A*	0.4037	0.057	-0.048

Summary of results from an association analyses for maternally inherited two-marker-haplotypes based on a model (1) including a fixed two-marker-haplotype effect and a random polygenic effect. Individual two-marker-haplotypes were compared to all other maternally inherited haplotypes in the data set. Haplotype frequencies were determined by direct counting.

## Discussion

In an approach to identify marker and marker haplotypes affecting SCS in the German Holstein population, we analysed association of marker genotypes and maternally inherited two-marker-intervals flanking putative QTL positions detected in an LALD analysis for SCS on BTA18, and showed that the two-marker-haplotype *249-G *of interval 12-13 (*BB710 *- *PVRL2_c.392G>A*) is in LD with SCS in the German Holstein population.

Initially, we detected a genome-wide significant QTL for SCS on BTA18 (Figure 1). The maxima of the QTL test statistic were in interval 9 (*HAMP_c.366+109G>A *- *BMS833*) and in interval 12 (*BB710 *- *PVRL2_c.-1268G>C*). In the same region telomeric on BTA18, several studies reported QTL for SCS. Kühn et al. [7], Brink [5] and Xu et al. [11] all identified a QTL for SCS in the German Holstein population at the telomeric end of BTA18 near marker *TGLA227*. Schulman et al. [10] reported a QTL for SCS as well as for mastitis at the telomeric end of BTA18 in Finnish Ayrshire cattle, and Ashwell et al. [50] localized a QTL for SCS in the middle to telomeric region on BTA18 at marker BM2078 in US Holstein, whereas Lund et al. [8] reported a QTL for SCS in Finnish Ayrshire, Swedish Red and White, and Danish Red with the maximum of the test statistic in the middle of BTA18 between the markers ILSTS002 and BMS2639. Interestingly Lund et al. [8] as well as Xu et al. [11] indicated that there might be more than one QTL for SCS on BTA18, but in both studies no significant evidence could be provided for a second QTL. Due to the lower marker density in previous studies and different approaches to detect QTL, a direct comparison between our results and previously mentioned studies is impeded. Nevertheless, the confidence intervals of the maxima observed in our LALD analysis did not include marker *TGLA227 *at the telomeric end of BTA18, and the QTL position reported by Lund et al. [8] as well as the assumption of a second QTL by Xu et al. [11] further in the middle of BTA18, indicated that we identified a second QTL for SCS in German Holstein cattle and possibly discovered a third QTL on BTA18 in our studies. Further indications for a third QTL on BTA18 in German Holstein cattle arise from association and haplotype analyses but no formal proof for a third QTL is given in our studies.

Subsequent to QTL mapping, we performed an association analysis of marker genotypes to verify the association of candidate gene polymorphisms and flanking markers of the most likely QTL positions observed in LALD analysis with SCS. Our results indicated that the microsatellite marker *BMS833 *(p = 0.004) is associated with SCS and *PVRL2_c.392G>A *(p = 0.017) and *CALM3_c.3+1678C>T *(p = 0.055) showed a respective tendency of association. Corresponding to the results of our LALD analysis, these results confirmed the position of one QTL for SCS in interval 9, as *BMS833 *is one of the flanking markers of this interval. *PVRL2_c.392G>A *and *CALM3_c.3+1678C>T *are flanking markers of the intervals 13 (*PVRL2_c.-1268G>C *- *PVRL2_c.392G>A*), 14 (*PVRL2_c.392G>A *- DIK3014) and intervals 16 (DIK4234 - *CALM3_c.3+1678C>T*) and 17 (*CALM3_c.3+1678C>T *- *CALM3_c.3+1795C>T*). The weak association observed for these markers is also in accordance to our LALD analysis, because interval 13 and 14 are within the genome-wide significance threshold in LALD analysis. Additionally, the second maximum observed in our LALD analysis in interval 12 is in an immediately adjacent interval and the putative QTL position in interval 12 is approximately 1 MB upstream of *PVRL2_c.392G>A*. For *CALM3_c.3+1678C>T*, only interval 16 (*DIK4234 *- *CALM3_c.3+1678C>T*) is within the chromosome-wide significance level in our LALD test statistic confirming the weaker association with SCS observed. To further test the results of our LALD analysis and the association of *BMS833 *genotypes with SCS on a population wide level, we investigated maternally inherited two-marker-intervals for interval 9 (*HAMP_c.366+109G>A *- *BMS833*) as well as for interval 12-13 (*BB710 *- *PVRL2_c.392G>A*). First we analysed effects of maternally inherited two-marker-interval gametes estimated based on IBD coefficients and second we performed a direct association analysis for maternally inherited two-marker-haplotypes of interval 9 and interval 12-13 without considering IBD coefficients.

For interval 9 (*HAMP_c.366+109G>A *- *BMS833*), maternally inherited two-marker-interval gametes showed differences in estimated effects for SCS (Figure 2). The variance of estimated gametic effects was higher for two-marker-interval gametes in interval 9 compared to two-marker-interval gametes in interval 12-13 and single marker analyses revealed that the microsatellite marker alleles are the main force in discriminating the effects of maternally inherited two-marker-interval gametes (Figure 2, Figure 3). Association analysis for maternally inherited two-marker-haplotypes of interval 9 showed that none of the maternally inherited two-marker-haplotypes in interval 9 (*HAMP_c.366+109G>A *- *BMS833*) are in LD with SCS in the German Holstein population. However, association analysis of *BMS833 *genotypes showed an association with SCS in our half-sib design indicating that the association is due to linkage but not linkage disequilibrium of the BMS833 locus with the causal mutation affecting SCS.

For interval 12-13 (*BB710 *- *PVRL2_c.392G>A*), the biggest difference in gametic effects estimated for maternally inherited two-marker-interval gametes was observed between *249-G *(0.0378 (± 0.0018)) and *253-A *(- 0.0253 (± 0.0012)), where a positive mean of estimates indicates an unfavourable effect on SCS (high number of cells) and a negative mean of estimates indicates a favourable effect on SCS (low number of cells). Association analyses for the maternally inherited two-marker-haplotypes of interval 12-13 showed that *249-G *(p = 0.027) is associated with SCS at the nominal 5% significance level in the German Holstein population. The weak association of *PVRL2_c.392G>A *genotype with SCS within the families we investigated and the association of maternally inherited two-marker-haplotype *249-G *with SCS indicates that *PVRL2_c.392G>A *is not the causal mutation affecting SCS in German Holstein cattle, but the causal mutation has to be located near or within marker interval 12-13 (*BB710 *- *PVRL2_c.392G>A*). Combining results obtained for markers *BMS833 *and *PVRL2_c.392G>A *it still remains unclear whether there are two mutations (two QTL), one located near marker *BMS833 *(interval 9) and one near or within interval 12-13, or only one mutation, presumably in interval 12-13, affecting SCS in the middle part of BTA18.

The candidate gene polymorphisms we investigated were not the causal mutations affecting SCS in German Holstein cattle. However, the results of association analyses for single marker genotypes and maternally inherited two-marker-haplotypes indicated that *HAMP *and *PVRL2 *were selected within the region harbouring at least one QTL for SCS. Particularly *PVRL2 *still remains interesting. On the one hand, the association of the *PVRL2_c.392G>A *genotype and that of the *249-G *haplotype suggests that another polymorphism within *PVRL2 *is the causal mutation affecting SCS in the German Holstein population. On the other hand, *PVRL2 *was selected as a gene with possible impact on mammary gland development or udder morphology and several studies have detected QTL for udder conformation on BTA18 [51,52]. Therefore, it is also possible that *PVRL2 *does not directly affect SCS but affects udder conformation traits like udder depth or fore udder attachment that are correlated with SCS [1].

## Conclusion

In summary, our results suggest that the chromosomal region including interval 9 (*HAMP_c.366+109G>A *- *BMS833*) and interval 12-13 (*BB710 *- *PVRL2_c.392G>A*), in the middle to telomeric region on BTA18 has a strong impact on SCS in the German Holstein population. The analyses of maternally inherited two-marker-interval gamete effects and the association of the maternally inherited two-marker-haplotype *249-G *of interval 12-13 (*BB710 *- *PVRL2_c.392G>A*) with SCS indicates that micosatellite marker *BB710 *could be a suitable candidate-marker for MAS, but association of microsatellite marker *BB710 *with SCS has to be verified. To confirm the association of the two-marker-haplotype *249-G *with SCS and approve the hypothesis of two QTL in this region a further investigation is necessary. Thus, a mapping of udder conformation traits including a multiple-trait and multiple-QTL model might be useful to verify the existence of two QTL, and whether they are both directly affecting SCS or one is affecting a correlated trait. Likewise, a higher marker density within this region has to be achieved and families segregating for different *BB710 *alleles have to be identified. Hence, it might be useful to cover the region including interval 9 and interval 12-13 with equally distributed SNP to narrow down the position of the casual mutation or mutations affecting SCS in the German Holstein population by a further fine mapping approach.

## Authors' contributions

BB carried out the genotyping work, the polymorphism screening, the linkage map construction, participated in the statistical analyses and drafted the manuscript. CB facilitated the statistical analyses by the development of software packages, participated in the statistical analyses and helped drafting the manuscript. MM participated in the development of software packages and the statistical analyses. NR participated in design and coordination of the study. CK devised the design of the study, coordinated the study, and participated in the statistical analyses and in drafting the manuscript. All authors read and approved the final manuscript.

## Supplementary Material

Additional file 1 **Marker table**. Summary of marker information including marker name, intervals, number of alleles, polymorphism information content, position of markers in own linkage map and in published linkage and RH maps and position in the bovine whole genome assemblies NCBI Build3.1 [GenBank: CM000194.3] and Btau4.0 [GenBank: NC_007316.3] as well as comparative position in the human whole genome assembly HSA36.3. Markers of intervals with marker spacing set to small values greater than zero are indicated by *. For some markers no accession number was available, therefore references for sequence information are given [5,53]. The polymorphism information content was calculated using the software PowerMarker v3.25 [54]. Marker positions are in order to positions of 5'-nucleotides of upstream primers for microsatellite markers or the direct position of the SNP. In Build3.1 several discrepancies in the sequence assembly were discovered. Discrepancies to the refined marker order are highlighted in yellow. Comparative positions in HSA36.3 were assigned by BLAST search of marker sequences in the bovine whole genome assembly Btau4.0 to identify the nearest gene locus on BTA18 and locating this gene locus in HSA36.3 using NCBI Map Viewer [34]. Click here for file

Additional file 2 **Primer table sequencing**. Summary of primers used for sequencing, including primer sequence, position in Btau4.0 and polymorphisms detected in PCR fragments. Click here for file

Additional file 3 **Primer table genotyping**. Summary of primers used for genotyping microsatellites, including primer sequence and position in Btau4.0. Click here for file

Additional file 4 **Polymorphisms**. Summary of polymorphisms identified by comparative sequencing including polymorphism name, accession number and sequence information for 200 nucleotides surrounding the polymorphism. Click here for file

## References

[B1] Rupp R, Boichard D (2003). Genetics of resistance to mastitis in dairy cattle. Vet Res.

[B2] Hinrichs D, Stamer E, Junge W, Kalm E (2005). Genetic analyses of mastitis data using animal threshold models and genetic correlation with production traits. J Dairy Sci.

[B3] Kalm E (2002). Development of cattle breeding strategies in Europe. Arch Tierz.

[B4] Khatkar MS, Thomson PC, Tammen I, Raadsma HW (2004). Quantitative trait loci mapping in dairy cattle: review and meta-analysis. Genet Sel Evol.

[B5] Brink M (2003). Ein Beitrag zur Feinkartierung von QTL-Regionen für Eutergesundheit beim Rind. Schriftenreihe des Instituts für Tierzucht und Tierhaltung der Christian-Albrechts-Universität zu Kiel.

[B6] Holmberg M, Andersson-Eklund L (2004). Quantitative Trait Loci Affecting Health Traits in Swedish Dairy Cattle. J Dairy Sci.

[B7] Kühn C, Bennewitz J, Reinsch N, Xu N, Thomsen H, Looft C, Brockmann GA, Schwerin M, Weimann C, Hiendleder S, Erhardt G, Medjugorac I, Forster M, Brenig B, Reinhardt F, Reents R, Russ I, Averdunk G, Blumel J, Kalm E (2003). Quantitative Trait Loci Mapping of Functional Traits in the German Holstein Cattle Population. J Dairy Sci.

[B8] Lund MS, Sahana G, Andersson-Eklund L, Hastings N, Fernandez A, Schulman N, Thomsen B, Viitala S, Williams JL, Sabry A, Viinalass H, Vilkki J (2007). Joint Analysis of Quantitative Trait Loci for Clinical Mastitis and Somatic Cell Score on Five Chromosomes in Three Nordic Dairy Cattle Breeds. J Dairy Sci.

[B9] Schrooten C, Bovenhuis H, Coppieters W, Van Arendonk JAM (2000). Whole Genome Scan to Detect Quantitative Trait Loci for Conformation and Functional Traits in Dairy Cattle. J Dairy Sci.

[B10] Schulman NF, Viitala SM, de Koning DJ, Virta J, Maki-Tanila A, Vilkki JH (2004). Quantitative Trait Loci for Health Traits in Finnish Ayrshire Cattle. J Dairy Sci.

[B11] Xu N, Paul S, Bennewitz J, Reinsch N, Thaller G, Reinhardt F, Kühn C, Schwerin M, Erhardt G, Weimann C, Thomsen H, Mishra S, Kalm E (2006). Confirmation of quantitative trait loci for somatic cell score on bovine chromosome 18 in the German Holstein. Arch Tierz.

[B12] Baes C, Brand B, Mayer M, Kuhn C, Liu Z, Reinhardt F, Reinsch N (2009). Refined positioning of a quantitative trait locus affecting somatic cell score on chromosome 18 in the German Holstein using linkage disequilibrium. J Dairy Sci.

[B13] Kühn C, Reinhardt F, Schwerin M (2008). Marker assisted selection of heifers improved milk somatic cell count compared to selection on conventional pedigree breeding values. Arch Tierz.

[B14] Pfitzer G (2001). Signal Transduction in Smooth Muscle: Invited Review: Regulation of myosin phosphorylation in smooth muscle. J Appl Physiol.

[B15] Walsh MP (1994). Calmodulin and the regulation of smooth muscle contraction. Mol Cell Biochem.

[B16] Park CH, Valore EV, Waring AJ, Ganz T (2001). Hepcidin, a Urinary Antimicrobial Peptide Synthesized in the Liver. J Biol Chem.

[B17] Peyssonnaux C, Zinkernagel AS, Datta V, Lauth X, Johnson RS, Nizet V (2006). TLR4-dependent hepcidin expression by myeloid cells in response to bacterial pathogens. Blood.

[B18] Ganz T (2007). Molecular Control of Iron Transport. J Am Soc Nephrol.

[B19] Ivanov DB, Philippova MP, Tkachuk VA (2001). Structure and Functions of Classical Cadherins. Biochemistry (Mosc).

[B20] Ebnet K (2008). Organization of multiprotein complexes at cell-cell junctions. Histochem Cell Biol.

[B21] Takai Y, Ikeda W, Ogita H, Rikitake Y (2008). The Immunoglobulin-Like Cell Adhesion Molecule Nectin and Its Associated Protein Afadin. Annu Rev Cell Dev Biol.

[B22] Niessen CM (2007). Tight Junctions/Adherens Junctions: Basic Structure and Function. J Invest Dermatol.

[B23] Ireton K (2007). Entry of the bacterial pathogen Listeria monocytogenes into mammalian cells. Cell Microbiol.

[B24] Pizarro-Cerdá J, Cossart P (2006). Bacterial Adhesion and Entry into Host Cells. Cell.

[B25] Hall TA (1999). BioEdit: a user-friendly biological sequence alignment editor and analysis program for Windows 95/98/NT. Nucleic Acids Symp Ser.

[B26] Liu Z, Reinhardt F, Bunger A, Reents R (2004). Derivation and Calculation of Approximate Reliabilities and Daughter Yield-Deviations of a Random Regression Test-Day Model for Genetic Evaluation of Dairy Cattle. J Dairy Sci.

[B27] Cattle Genome Mapping Project. http://www.marc.usda.gov/genome/cattle/cattle.html.

[B28] Vincze T, Posfai J, Roberts RJ (2003). NEBcutter: a program to cleave DNA with restriction enzymes. Nucl Acids Res.

[B29] NEBcutter V2.0. http://tools.neb.com/NEBcutter2/index.php.

[B30] Green P, Falls K, Crooks S (1990). Documentation of CRI-MAP, Version 24.

[B31] Snelling W, Casas E, Stone R, Keele J, Harhay G, Bennett G, Smith T (2005). Linkage mapping bovine EST-based SNP. BMC Genomics.

[B32] Ihara N, Takasuga A, Mizoshita K, Takeda H, Sugimoto M, Mizoguchi Y, Hirano T, Itoh T, Watanabe T, Reed KM, Snelling WM, Kappes SM, Beattie CW, Bennett GL, Sugimoto Y (2004). A Comprehensive Genetic Map of the Cattle Genome Based on 3802 Microsatellites. Genome Res.

[B33] Itoh T, Watanabe T, Ihara N, Mariani P, Beattie CW, Sugimoto Y, Takasuga A (2005). A comprehensive radiation hybrid map of the bovine genome comprising 5593 loci. Genomics.

[B34] NCBI Map Viewer. http://www.ncbi.nlm.nih.gov/mapview/.

[B35] Rexroad CE, Owens EK, Johnson JS, Womack JE (2000). A 12 000 rad whole genome radiation hybrid panel for high resolution mapping in cattle: characterization of the centromeric end of chromosome 1. Anim Genet.

[B36] Boehnke M, Lunetta K, Hauser E, Lange K, Uro J, VanderStoep J (1996). RHMAP: Statistical Package for Multipoint Radiation Hybrid Mapping Version 30.

[B37] Baes C, Reinsch N (2008). TIGER: A software system for fine-mapping quantitative trait loci. Arch Tierz.

[B38] Reinsch N (1999). A multiple-species, multiple-project database for genotypes at codominant loci. J Anim Breed Genet.

[B39] Meuwissen THE, Goddard ME (2000). Fine mapping of quantitative trait loci using linkage disequilibria with closely linked marker loci. Genetics.

[B40] Meuwissen THE, Goddard ME (2001). Prediction of identity by descent probabilities from marker-haplotypes. Genet Sel Evol.

[B41] Meuwissen THE, Karlsen A, Lien S, Olsaker I, Goddard ME (2002). Fine mapping of a quantitative trait locus for twinning rate using combined linkage and linkage disequilibrium mapping. Genetics.

[B42] Baes C, Reinsch N (2007). Computing the condensed conditional gametic QTL relationship matrix and its inverse. Arch Tierz.

[B43] Gilmour AR, Gogel BJ, Cullis BR, Thompson R (2006). ASReml User Guide Release 20.

[B44] Lander ES, Botstein D (1989). Mapping Mendelian Factors Underlying Quantitative Traits Using Rflp Linkage Maps. Genetics.

[B45] Ott J (1991). Analysis of Human Genetic Linkage. Revised edition.

[B46] NCBI HomePage. http://www.ncbi.nlm.nih.gov/.

[B47] NCBI BLAST. http://blast.ncbi.nlm.nih.gov/Blast.cgi.

[B48] NCBI Trace Archives. http://www.ncbi.nlm.nih.gov/Traces/home/.

[B49] Elsik CG, Tellam RL, Worley KC, The Bovine Genome Sequencing and Analysis Consortium (2009). The Genome Sequence of Taurine Cattle: A Window to Ruminant Biology and Evolution. Science.

[B50] Ashwell M, Rexroad CEJ, Miller R, Vanraden P, Da Y (1997). Detection of loci affecting milk production and health traits in an elite US Holstein population using microsatellite markers. Anim Genet.

[B51] Kolbehdari D, Wang Z, Grant JR, Murdoch B, Prasad A, Xiu Z, Marques E, Stothard P, Moore SS (2008). A Whole-Genome Scan to Map Quantitative Trait Loci for Conformation and Functional Traits in Canadian Holstein Bulls. J Dairy Sci.

[B52] Schnabel RD, Sonstegard TS, Taylor JF, Ashwell MS (2005). Whole-genome scan to detect QTL for milk production, conformation, fertility and functional traits in two US Holstein families. Anim Genet.

[B53] Thomsen H, Reinsch N, Xu N, Looft C, Grupe S, Kuhn C, Brockmann GA, Schwerin M, Leyhe-Horn B, Hiendleder S, Erhardt G, Medjugorac I, Russ I, Forster M, Brenig B, Reinhardt F, Reents R, Blumel J, Averdunk G, Kalm E (2000). A male bovine linkage map for the ADR granddaughter design. J Anim Breed Genet.

[B54] Liu KJ, Muse SV (2005). PowerMarker: an integrated analysis environment for genetic marker analysis. Bioinformatics.

